# An R2R3 MYB transcription factor associated with regulation of the anthocyanin biosynthetic pathway in Rosaceae

**DOI:** 10.1186/1471-2229-10-50

**Published:** 2010-03-21

**Authors:** Kui Lin-Wang, Karen Bolitho, Karryn Grafton, Anne Kortstee, Sakuntala Karunairetnam, Tony K McGhie, Richard V Espley, Roger P Hellens, Andrew C Allan

**Affiliations:** 1 The New Zealand Institute for Plant & Food Research Ltd, (Plant and Food Research), Mt Albert Research Centre, Private Bag 92169, Auckland, New Zealand; 2 Wageningen UR Plant Breeding, Postbus 386, 6700 AJ, Wageningen, The Netherlands; 3 Plant and Food Research, Palmerston North 4442, New Zealand

## Abstract

**Background:**

The control of plant anthocyanin accumulation is via transcriptional regulation of the genes encoding the biosynthetic enzymes. A key activator appears to be an R2R3 MYB transcription factor. In apple fruit, skin anthocyanin levels are controlled by a gene called *MYBA *or *MYB1*, while the gene determining fruit flesh and foliage anthocyanin has been termed *MYB10*. In order to further understand tissue-specific anthocyanin regulation we have isolated orthologous *MYB *genes from all the commercially important rosaceous species.

**Results:**

We use gene specific primers to show that the three MYB activators of apple anthocyanin (*MYB10/MYB1/MYBA) *are likely alleles of each other. MYB transcription factors, with high sequence identity to the apple gene were isolated from across the rosaceous family (e.g. apples, pears, plums, cherries, peaches, raspberries, rose, strawberry). Key identifying amino acid residues were found in both the DNA-binding and C-terminal domains of these MYBs. The expression of these *MYB10 *genes correlates with fruit and flower anthocyanin levels. Their function was tested in tobacco and strawberry. In tobacco, these MYBs were shown to induce the anthocyanin pathway when co-expressed with bHLHs, while over-expression of strawberry and apple genes in the crop of origin elevates anthocyanins.

**Conclusions:**

This family-wide study of rosaceous R2R3 MYBs provides insight into the evolution of this plant trait. It has implications for the development of new coloured fruit and flowers, as well as aiding the understanding of temporal-spatial colour change.

## Background

The Rosaceae is an economically important group of cultivated plants, which includes fruit-producing genera such as *Malus *(apples), *Pyrus *(pears), *Prunus *(e.g. peach, plums, apricots), *Fragaria *(strawberries), and *Rubus *(raspberry, blackberry, boysenberry), as well as ornamental plants such as *Rosa *(rose). In these fruits and flowers, colour is a key quality trait and is often caused by anthocyanin. Anthocyanins are water-soluble pigments that belong to the flavonoid family of compounds giving red, blue and purple colours in a range of flowers, fruits, foliage, seeds and roots [1]. Anthocyanins are involved in a wide range of functions, such as the attraction of pollinators, seed dispersal, protection against UV light damage, and pathogen attack [2-5]. Recently, research on anthocyanins has intensified because of their potential benefits to human health, including protection against cancer, inflammation, coronary heart diseases and other age-related diseases [6-11].

In plants, the structural genes of the flavonoid biosynthetic pathway are largely regulated at the level of transcription. In all species studied to date, the regulation of the expression of anthocyanin biosynthetic genes are through a complex of MYB transcription factors (TF), basic helix-loop-helix (bHLH) TFs and WD-repeat proteins (the MYB-bHLH-WD40 "MBW" complex; [12]). A model has been proposed for the activation of structural pigmentation genes, with regulators interacting with each other to form transcriptional complexes in conjunction with the promoters of structural genes [13]. For example, the R2R3 MYB C1 protein, that regulates the anthocyanin pathway in maize, interacts with a bHLH TF (either of the genes termed *B *or *R*) to activate the promoter of dihydroflavonol reductase (DFR). In contrast, the R2R3 MYB P protein, which regulates the phlobaphene pathway in maize, can activate the same promoter without a bHLH TF [14].

MYB TFs can be classified into three subfamilies based on the number of highly conserved imperfect repeats in the DNA-binding domain including R3 MYB (MYB1R) with one repeat, R2R3 MYB with two repeats, and R1R2R3 MYB (MYB3R) with three repeats [15,16]. Among these MYB transcription factors, R2R3-MYBs constitute the largest TF gene family in plants, with 126 R2R3 MYB genes identified in *Arabidopsis *[17]. Those associated with up-regulation of the anthocyanin pathway are R2R3 MYBs. Over-expression of the At*PAP1 *gene (At*MYB75, At1 g56650*) results in the accumulation of anthocyanins in *Arabidopsis *[18]. Several repressors of the phenylpropanoid pathway, and perhaps anthocyanins specifically, are also MYB TFs, including an R2R3 MYB repressor from strawberry Fa*MYB1 *[19], *Arabidopsis *At*MYB6*, *4*, and *3 *[20], *Antirrhinum *Am*MYB308 *[21], and a one repeat MYB in *Arabidopsis*, At*MYBL2 *[22,23]. How the repressor MYBs interact with the MBW transcriptional complex is beginning to be elucidated [22,23].

Based on the phylogenetic relationship between *Arabidopsis *R2R3 MYB TFs and anthocyanin-related MYBs of other species, it appears that anthocyanin-regulating R2R3 MYBs fall into one or two clades [17,24,25]. Anthocyanin-regulating MYBs have been isolated from many species, including *Arabidopsis *At*MYB75 *or *PAP1*, At*MYB90 *or *PAP2*, At*MYB113 *and At*MYB114 *[26], *Solanum lycopersicum ANT1 *[27], *Petunia hybrida AN2 *[28], *Capsicum annuum A *[29], *Vitis vinifera *Vv*MYB1a *[30], *Zea mays P *[31], *Oryza saliva C1 *[32], *Ipomoea batatas *Ib*MYB1 *[33], *Anitirrhinum majus ROSEA1, ROSEA2 *and *VENOSA *[34], *Gerbera hybrid *Gh*MYB10 *[35], *Picea mariana MBF1 *[36], *Garcinia mangostana *Gm*MYB10 *[37], *Malus *× *domestica *Md*MYB10*, Md*MYB1*/Md*MYBA *[24,38,39], and *Gentian *Gt*MYB3 *[40].

For rosaceous species, MYBs that regulate the genes of the anthocyanin pathway have been examined in apple and strawberry. In apple (*Malus *× *domestica*) *MYB10 *was isolated from red-fleshed apple 'Red Field' [24], and showed a strong correlation between the expression of *MYB10 *and apple anthocyanin levels during fruit development. Transgenic apple lines constitutively expressing *MYB10 *produced highly pigmented shoots. Two more apple TFs, *MYB1 *and *MYBA*, were also reported to regulate genes in the anthocyanin pathway in red-skinned fruit [38,39]. Both *MYB1 *and *MYBA *share identical sequences [38], while *MYB10 *and *MYB1 *genes are located at very similar positions on linkage group 9 of the apple genetic map [41]. In strawberry (*Fragaria *× *ananassa*), the R2R3 MYB TF Fa*MYB1 *plays a key role in down-regulating the biosynthesis of anthocyanins and flavonols [19].

In this current study, we used an allele-specific PCR primer approach to show that *MdMYB1/MdMYBA/MdMYB10 *are highly likely to be allelic in the apple genome. We then isolated genes with high sequence similarity to *MYB10 *from 20 species within the Rosaceae. Sequence and functional characterization of these genes provides insight into the evolution of this TF, within a plant family where higher levels of pigmentation has been selected for during the process of domestication. Expression analysis during the fruit development, and functional testing using transient assays and transgenic plants suggest that these R2R3 MYBs are responsible for controlling anthocyanin biosynthesis in these crops.

## Results

### The *MdMYB10/MdMYB1/MdMYBA *genes are likely to be allelic

**Figure 1:**
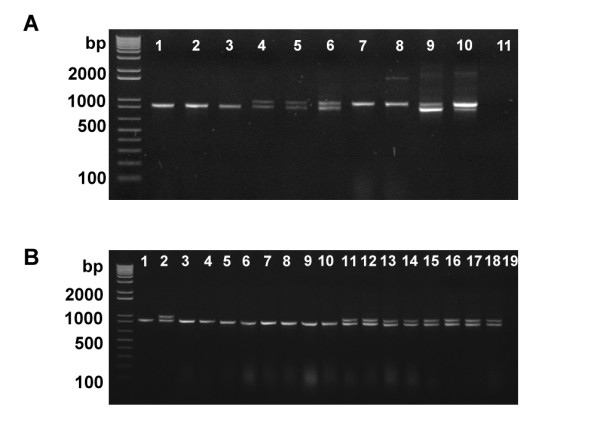
**Analysis of apple MYB10/MYB1 in diverse apple cultivars**. (A) Homozygous R1 MYB10 Pacific Rose™ (1), 'Royal Gala' (2), 'Granny Smith' (3); Heterozygous 'Red Field' OP (4), Niedzwetzkyana (5), 'Roberts Crab' (6); Homozygous R6 MYB10 *Malus sieversii *01P22 (7), *Malus sieversii *629319 (8); Mixture of diluted (1 to 10^6^) PCR products R1:R6 (3:1) (9), R1:R6 (1:3) (10); no template control (11). (B) Analysis of apple MYB10/MYB1 in Pacific Rose™ (lane 1) × 'Red Field' (lane 2) & segregation of progeny (lanes 3 to18). Lane 19 is no template control.

Three highly homologous apple genes, *MYB10 *[24], *MYB1 *[39] and *MYBA *[38], have been reported in different cultivars of apple. In order to ascertain whether, in any given cultivar, these represent different genes or are alleles of the one gene, we designed PCR primers to amplify a region of genomic DNA common to all three of these genes, spanning a region from the promoter through to exon 1 of the published sequences. This region produces an amplification length polymorphism distinguishing the *MYB10 *allele present in red-fleshed cultivars from white fleshed types [42]. The amplification products from a range of apple varieties are shown in Figure 1A. One amplification product of approximately 900 bp is observed for the white-fleshed varieties Pacific Rose™, 'Royal Gala', and 'Granny Smith'. Two amplification products, of approximately 900 bp and 1000 bp, were observed in red-fleshed apple varieties such as 'Red Field', 'Niedzwetzkyana', and 'Robert's Crab'. With red-fleshed varieties, known to be homologous for the red-flesh gene [41,42], only the 1000-bp fragment is amplified. These products represent the R_1 _and R_6 _alleles previously reported for *MYB10 *[42], and suggests that *MYB10 *and *MYB1 *are alleles, because if they were paralogues there would still be two products in R_6_R_6 _homozygous apples.

While these end-point PCR amplifications are not quantitative, the fluorescence from ethidium bromide (EtBr) indicated that in those tissues where both 900- and 1000-bp fragments are amplified, these molecules are likely to be in equivalent molar quantity within the genome. This is based on the observation that when a mixture of diluted PCR products from the 900-bp and 1000-bp fragments are mixed in ratios of 3:1 or 1:3 respectively, the EtBr fluorescence of the end-point PCR amplifications reflects the corresponding molar ratios (Figure 1A). Furthermore, PCR analysis of the progeny from crosses made between the R_1 _homozygous Pacific Rose™ cultivar and the heterozygous R_1_R_6 _'Red Field' shows segregation of the homozygous R_1 _allele and the heterozygous R_1 _and R_6 _alleles (Figure 1B). If *MYB1 *and *MYB10 *were different genes, band intensity ratios of 3:1 would be possible but as only 1:1 ratios are observed, *MYB1 *and *MYB10 *are likely to be allelic, representing the R_1 _and R_6 _alleles.

### Isolation of MYB10 homologues from the major rosaceous crop species

**Figure 2:**
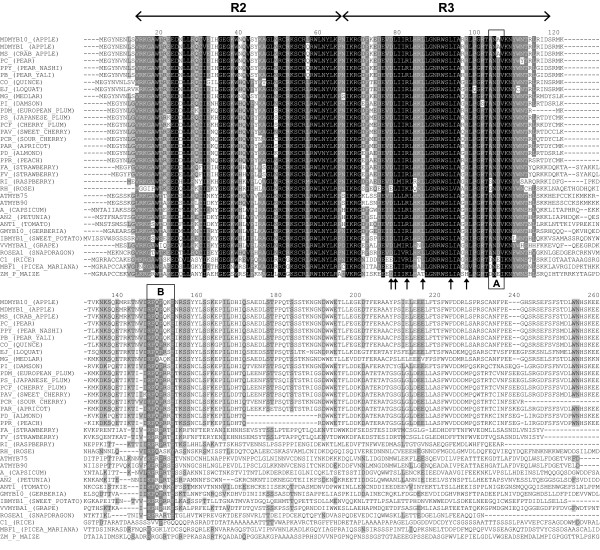
**Protein sequence alignment of rosaceous MYB10 and known anthocyanin MYB regulators from other species**. Arrows indicate specific residues that contribute to a motif implicated in bHLH co-factor interaction in *Arabidopsis *[44]. Box (A) a conserved motif [A/S/G]NDV in the R2R3 domain for dicot anthocyanin-promoting MYBs. Box (B) a C-terminal-conserved motif KPRPR [S/T]F for *Arabidopsis *anthocyanin-promoting MYBs [17].

We isolated both cDNA and genomic DNA from 20 rosaceous species and, using a gene-specific primer approach based on the apple *MYB10 *gene sequence, generated PCR fragments for cloning into sequencing vectors. Fragments with sequence similarity to *MYB10 *were used to obtain full-length sequences for further functional testing. This approach worked well for all the members of the *Maloideae *subfamily (including apple, quince, loquat, medlar and pear) and *Amygdaloideae *subfamily (including apricot, damson, cherry, plum, almond and peach), but not for species of the *Rosoideae *subfamily (rose, strawberry and raspberry). For *Rosoideae*, we required additional steps involving 5' and 3' GeneRace of mRNA (GeneRacer Kit, Invitrogen), with degenerate primers designed to the consensus DNA sequence of the anthocyanin-related R2R3 MYB DNA binding domain. The rosaceous MYB transcription factors isolated, using these approaches, are shown in Table 1, and predicted protein sequence is shown in Figure 2.

**Table 1:** Anthocyanin activating R2R3 MYBs transcription factors

*Species*	Current name	Genebank number	% similarity to AtMYB75 protein	% identity to AtMYB75	gDNA (bp)	CDS (bp)	protein (aa)	Intron2 (bp)
*Arabidopsis thaliana*	PAP1 AtMYB75	AF325123	100	100	1376	747	248	89
*Arabidopsis thaliana*	PAP2 AtMYB90	NM_105310	88	84	1349	750	249	82
*Solanum lycopersicum *(tomato)	ANT1	AY348870	56	41	n/a	825	274	n/a
*Petunia hybrida*	AN2	EF423868	66	45	n/a	768	255	n/a
*Capsicum annuum*	*A*	AJ608992	64	44	n/a	789	262	n/a
*Vitis vinifera *(grape)	VvMYB1a	AB242302	58	43	n/a	753	250	n/a
*Zea mays *(Maize)	P	AF292540	32	26	n/a	1131	376	n/a
*Oryza sativa *(Rice)	C1	Y15219	54	33	n/a	819	272	n/a
*Ipomoea batatas *(Sweet potato)	IbMYB1	AB258985	61	44	1194	750	249	313
*Antirrhinum majus *(snapdragon)	ROSEA1	DQ275529	66	52	n/a	663	220	n/a
*Gerbera hybrid*	GMYB10	AJ554700	58	44	n/a	753	250	n/a
*Picea mariana*	MBF1	PMU39448	30	41	n/a	1167	388	n/a
*Malus domestica *(apple)	MdMYB10	EU518249	60	47	4050	729	243	2995
*Malus domestica *(apple)	MdMYB1	DQ886414	60	47	4055	732	243	3000

*Malus sylvestris *(crab apple)	MsMYB10	EU153573	60	47	4036	732	243	2981
*Cydonia oblonga *(quince)	CoMYB10	EU153571	61	47	2436	738	245	1418
*Eriobotrya japonica *(loquat)	EjMYB10	EU153572	59	47	1520	741	246	498
*Mespilus germanica *(medlar)	MgMYB10	EU153574	60	47	2232	738	245	1168
*Pyrus communis *(Pear)	PcMYB10	EU153575	60	47	1545	735	244	487
*Pyrus pyrifolia *(Nashi)	PpyMYB10	EU153576	60	47	1541	735	244	483
*Pyrus × bretschneideri *(Chinese pear)	PbMYB10	EU153577	60	47	1546	735	244	488
*Prunus armeniaca *(Apricot)	ParMYB10	EU153578	*61*	49	2245	732	243	1211
*Prunus insititia *(Damson)	PiMYB10	EU153579	*62*	49	1924	732	242	882
*Prunus domestica *(European plum)	PdmMYB10	EU153580	*60*	48	2012	714	237	993
*Prunus avium *(sweet cherry)	PavMYB10	EU153581	*61*	50	2223	735	244	1123
*Prunus cerasus *(sour cherry)	PcrMYB10	EU153582	*64*	46	2291	678	225	1196
*Prunus cerasifera *(cherry plum)	PcfMYB10	EU153583	*61*	49	1960	732	243	926
*Prunus dulcis *(almond)	PdMYB10	EU155159	*61*	46	1796	678	225	812
*Prunus persica *(peach)	PprMYB10	EU155160	*60*	46	1845	675	224	947
*Prunus salicina *(Japanese plum)	PsMYB10	EU155161	*60*	49	1880	732	243	842
*Fragaria × ananassa *(strawberry)	FaMYB10	EU155162	*62*	45	1685	702	233	899
*Fragaria vesca *(strawberry)	FvMYB10	EU155163	*62*	44	1714	705	235	926
*Rosa hybrida *(rose)	RhMYB10	EU155164	*59*	40	1122	750	249	264
*Rubus idaeus *(red raspberry)	RiMYB10	EU155165	*58*	43	1685	654	217	806

MYB transcription factors, homologous to apple MdMYB10, from all the major rosaceous species (below the middle line), and the published anthocyanin MYB regulators from other species (above the middle line).

For both protein sequence and coding DNA sequence (CDS) of rosaceous MYBs, the percentage of identity to *Arabidopsis *At*MYB75 *(*PAP1*, AT1G56650) varied from 58 to 64%, and 40 to 49%, respectively. The length of CDS and protein sequence was similar between each species analysed, but the length of genomic DNA (gDNA) sequence varied significantly from 1122 bp (*Rosa hybrida) *to 4055 bp (*Malus *× *domestica*, Table 1). This is due almost entirely to the variable length of intron 2, which ranges from 82 bp (*AtMYB90*) to 3000 bp (*MdMYB1*). A schematic of *MYB10*-like genes from rosaceous species is shown in Additional File 1. The large size of intron 2 in apple correlates with its higher DNA content than close relatives; apple has almost 2.5 times more DNA mass than pear [43 ]http://www.kew.org/cval/homepage.html. Intron 2 of apple *MYB10 *is 2995 bp, compared with 487 bp in pear (Additional File 1B).

**Figure 3:**
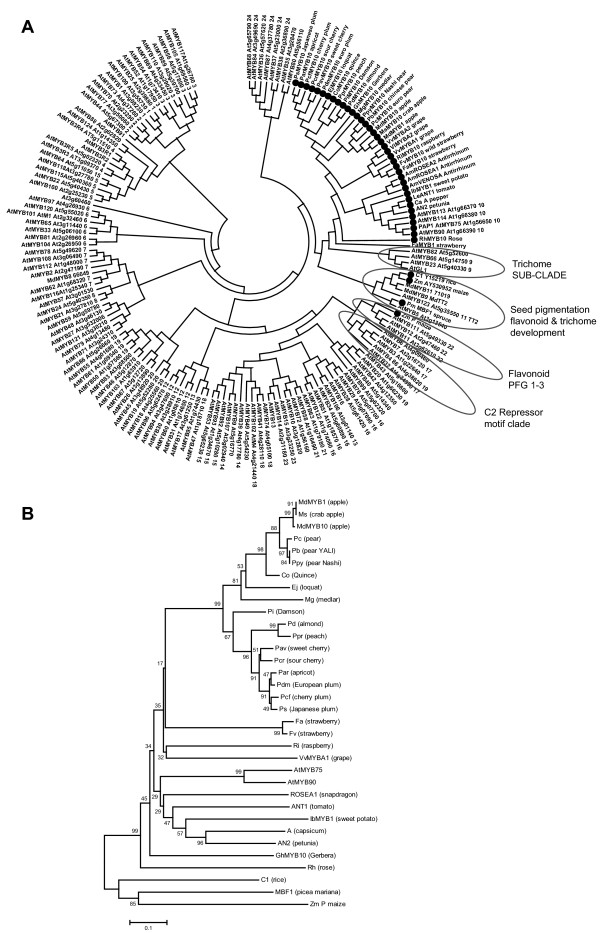
**Phylogenetic relationships between *Arabidopsis *MYB transcription factors and anthocyanin-related MYBs of rosaceous and other species**. Rosaceous MYB10s cluster next to PAP1 (AtMYB75) and PAP2 (AtMYB90), within the anthocyanin MYB regulator subgroup (A). A Phylogeny of MYB10 from all the major rosaceous species and known anthocyanin MYB regulators from other species (B). Sequences were aligned using Clustal W (opening = 15, extension = 0.3) in Vector NTI 9.0. Phylogenetic and molecular evolutionary analysis was conducted using MEGA version 3.1 [80] [using minimum evolution phylogeny test and 1000 bootstrap replicates].

When the region of homology, corresponding to the MYB R2R3 domain, was used to generate a phylogenic tree, all the genes clustered with known anthocyanin-related MYBs (Figure 3A). Furthermore, the MYB genes clustered according to their taxonomic relationships in the Rosaceae (Figure 3B). For the *Maloideae *(apple, pear, quince, loquat and medlar), all clustered together into a clade. For the *Amygdaloideae *(plum, cherry, almond, apricot, peach and damson), all were clustered into another clade. Raspberry, strawberry and rose are the members of the *Rosoideae *and they all clustered together. While the *Maloideae *and *Amygdaloideae *clustered closely together, the *Rosoideae *clustered more distantly.

### Sequence signatures specific for anthocyanin-related MYBs

The large gene family of R2R3 MYB proteins was examined using conserved regions of homology. Over 172 proteins were included; all *Arabidopsis *R2R3 MYBs, 38 other dicot anthocyanin-promoting MYBs, including apple MYB8, MYB9 and MYB11 (GenBank DQ267899, DQ267900, and DQ074463 respectively), strawberry anthocyanin repressor MYB1, as well as anthocyanin-related MYBs from four monocots and one gymnosperm. All the MYBs associated with promoting anthocyanin biosynthesis from dicot species cluster within the same clade as PAP1 and other *Arabidopsis *MYBs of this subgroup (Figure 3A). Monocot sequences, such as C1 and P, as well as the gymnosperm *Picea mariana *MBF1, cluster outside this group, suggesting that this clade is dicot-specific. The function of promoting anthocyanin biosynthesis for this subgroup may therefore have evolved after the divergence between dicots and monocots.

**Figure 4:**
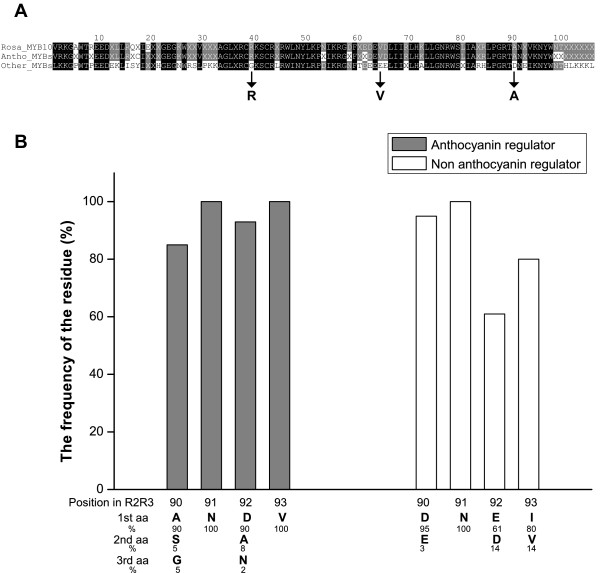
**Analysis of R2R3 DNA binding domains of anthocyanin-promoting MYBs**. Alignment (A) of three consensus amino-acid sequences from 22 rosaceous MYB10s, 38 dicot anthocyanin-promoting MYBs, and the other 134 proteins included in Figure 3A. To obtain three consensus sequences, the sequences in each of three groups were aligned using AlignX (opening = 15, extension = 0.3) in Vector NTI 9.0, and residue fraction for consensus was set to 0.9 for the alignments of 22 rosaceous MYB10s and 38 dicot anthocyanin-promoting MYBs, and 0.3 for the alignment of the other 134 proteins. (B) Frequency of residues at position 90 to 93 of the R2R3 domain covering 168 MYB TFs of *Arabidopsis*, rosaceous species, and other dicot sequences.

To ascertain if there is an identifiable protein motif specific for anthocyanin-promoting MYBs in the N-terminal R2R3 domain, the isolated rosaceous MYBs and other anthocyanin-promoting MYBs (16 from other dicot species) were compared with 134 MYB peptide sequences of other clades (Figure 4). Three amino-acid residues (arginine (R), valine (V), alanine (A); marked with arrows in Figure 4A) are conserved for dicot anthocyanin-promoting MYBs at a frequency of 100(R):92(V):90(A). None of these amino-acid residues appeared in the other 134 sequences at the respective position (full dataset in Additional File 2). Another convenient identifier for an anthocyanin-promoting MYB appears to be ANDV (in over 90% of cases) at position 90 to 93 in the R2R3 domain (Figure 2 Box A and Figure 4B) which is not seen in any other R2R3 MYBs (Additional File 2).

Outside of the DNA-interacting R2R3 domain, most R2R3 MYB proteins have a long C-terminal sequence. In this region of *Arabidopsis *anthocyanin-promoting MYBs, the motif KPRPR [S/T]F has been identified (Box B in Figure 2) [17], which is not present in other R2R3 MYBs. When anthocyanin-promoting MYB sequences from other species are aligned, this C-terminus consensus motif was still identifiable but with slight variations (Figure 2) to become [R/K]Px [P/A/R]xx [F/Y]. Within the subfamilies *Maloideae *and *Amygdeloideae*, there was over 70% similarity of C-terminus. An 18 amino acid deletion occurred in the C-terminus of both almond and peach (Figure 2) which is within exon 3, indicating that this is not a mis-prediction of an exon-intron boundary. However, this deletion did not disrupt the activity of peach MYB10 (see next section). Other anthocyanin-related MYBs are known to repress the biosynthetic pathway (e.g., FaMYB1, AtMYB3, AtMYBL2). These contain C-terminal motifs such as the ERF-associated amphiphilic repression (EAR) motif or the TLLLFR motif [22,23]. Such motifs were not found in any of the MYB10-like predicted proteins identified in this study.

A conserved amino acid signature ([D/E]Lx_2 _[R/K]x_3_Lx_6_Lx_3_R) (the locations indicated by the arrows in Figure 2) has been shown to be functionally important for the interaction between MYB and R/B-like bHLH proteins [44]. All rosaceous MYB sequences, as well as anthocyanin-related dicot MYBs and PmMBF1 and C1 had this signature. However, other R2R3 MYB TFs also have this signature (e.g., *Arabidopsis *MYBs TT2 [12] and AtMYBL2 [45]). Therefore, the presence of this motif is not indicative of the candidate MYB being within the anthocyanin-promoting clade, but rather suggests that these MYBs require an interacting bHLH partner.

### Functional assay of rosaceous MYB activity

Transient luciferase assays in the tobacco species *Nicotiana benthamiana *have been used to assay MYB activity against the *Arabidopsis *DFR-promoter (dihydro flavanoid reductase; At5g42800, [24,46]). Full length cDNAs of apple (*MYB10*), wild and cultivated strawberry (Fv and Fa*MYB10*), rose (Rh*MYB10*) and raspberry (Ri*MYB10*), and genomic DNA of pear, European plum, cherry-plum, cherry, apricot, and peach (Pc*MYB10*, Pdm*MYB10*, Pcf*MYB10*, Pav*MYB10*, Par*MYB10*, and Ppr*MYB10*, respectively) were cloned into the transient expression vector pGreen II 0024 62K [46] and transfected into *Agrobacterium*. These TFs were then co-infected into *N. benthamiana *leaves with AtDFR-LUC in a second *Agrobacterium *strain, with or without a bHLH co-factor in a third *Agrobacterium *strain. Trans-activation was assayed 3 days later as a change in LUC/REN ratio.

**Figure 5:**
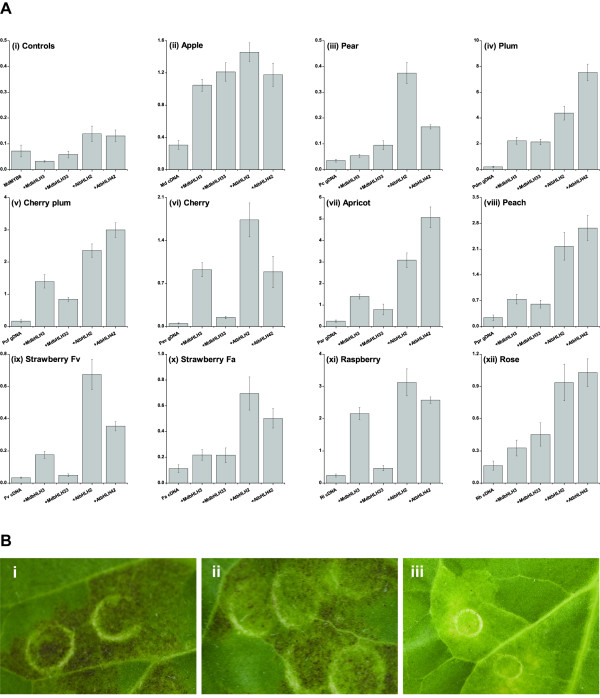
**Transient activation of anthocyanic responses by rosaceous MYB10s and bHLH transcription factors**. (A) Activation of the *Arabidopsis *DFR promoter by MYB10 and bHLH transcription factors. Error bars are the SE for eight replicate reactions. (B) Patches of anthocyanin production in tobacco leaves by PdmMYB10 (i), PprMYB10, but not by the negative control MdMYB8 (iii).

As shown in Figure 5A, all 11 MYB10s induced the DFR promoter, but only in the presence of a bHLH partner (either At*bHLH2*, At*bHLH42*, Md*bHLH3 *or Md*bHLH33*). In all cases, *MYB10 *activity increased to the greatest extent with At*bHLH2 *or At*bHLH42*. Apple *MYB10 *performed well with apple *bHLHs*. With cherry-plum, European plum, apricot, and raspberry, the induction by the MYB and bHLH was highly efficient, out-performing 35S:*Renilla *by at least 3-fold. Some of the MYB10 TFs (e.g., strawberry, pear, peach and rose) performed poorly with Md*bHLH3*. The poorest activator of At*DFR-LUC*, Pc*MYB10*, could enhance transcription of the LUC reporter to 0.45 of 35S:*Renilla *with At*bHLH2 *as a partner. *MYB8*, an apple R2R3 MYB from an unrelated clade, was included as a negative control. The induction of At*DFR-LUC *by Md*MYB8*, with At*bHLH2*, At*bHLH42*, Md*bHLH3 *or Md*bHLH33*, was significantly lower than all rosaceous MYB10s.

As previously reported [24] a patch of foliar anthocyanin production can be induced in *Nicotiana tabacum *leaves by co-expression of Md*MYB10 *with Md*bHLH3*. Induction of anthocyanin biosynthesis in transient assays by rosaceous MYB10s was tested and found to be dependent on the co-expression of the bHLH proteins from *Arabidopsis *or apple. Patches of anthocyanin were most apparent with Pdm*MYB10 *and Ppr*MYB10 *when At*bHLH2 *was included as a partner (Figure 5B).

### Expression of rosaceous MYB10 TFs correlate with anthocyanin biosynthesis

**Figure 6:**
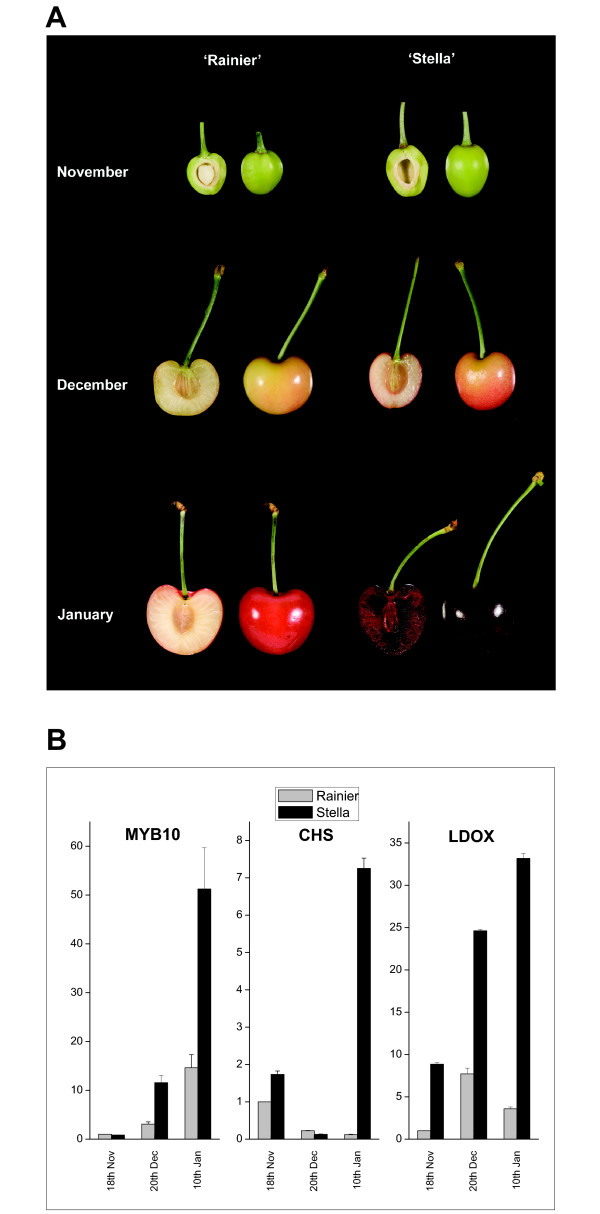
**Normalized quantitative Real-Time of the expression of cherry *PavMYB10***. Expression of PavMYB10 n the developmental series from sweet cherry 'Rainier' and 'Stella'. (A) Fruit sampled and (B) qPCR expression of *PavMYB10, CHS *and *LDOX *using 'Rainier' green fruitlet as a calibrator. Error bars are the SE for three replicate reactions.

Expression of sweet cherry *PavMYB10 *gene transcript was examined using qPCR analysis during fruit development in two cherry cultivars, 'Rainier' and 'Stella'. These two cultivars differ in the level of anthocyanin that accumulates in mature fruit (Figure 6A). At maturity, 'Rainier' appears pink as anthocyanin accumulates in the fruit skin, while 'Stella' is a deep red variety with high skin and flesh anthocyanin at maturity. Transcript of *PavMYB10 *accumulated in the fruit tissues of both cultivars. However, the level of expression is much higher in the fruit of 'Stella' compared with 'Rainier' at the latter two stages of fruit development (Figure 6B). Expression of cherry *CHS*, an early step in the anthocyanin biosynthesis pathway, and cherry *LDOX*, a later step, showed up-regulation correlated with cherry colour (Figure 6B).

**Figure 7:**
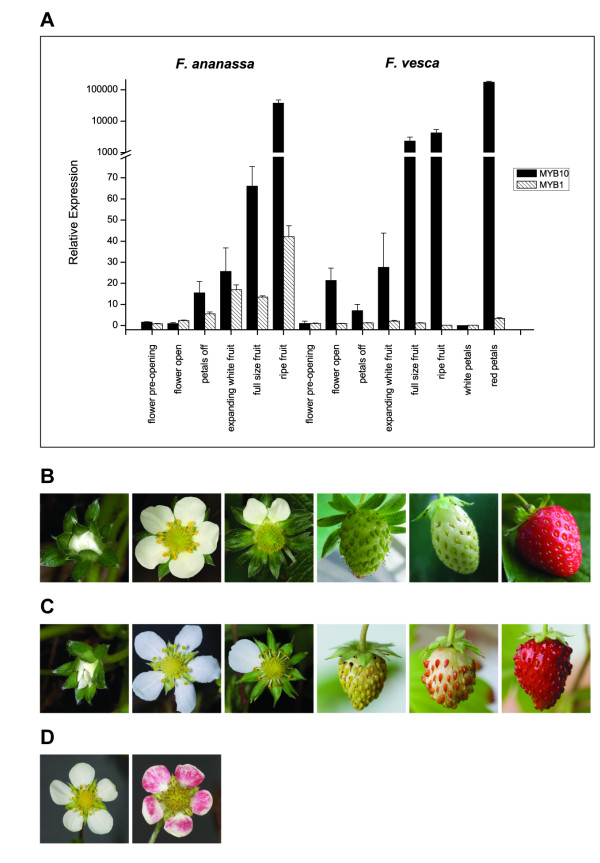
**Normalized qPCR data of the expression of strawberry *MYB10 *and *MYB1***. qPCR expression of *Fragaria MYB10 *and *MYB1 *in a fruit developmental series from both cultivated and wild strawberry (A). Fv stage 1 was set as a calibrator. Error bars are the SE for three replicate reactions. (B) Six developmental stages of cultivated strawberry. (C) Six developmental stages of wild strawberry. (D) White and red petals of wild strawberry.

Expression of the strawberry genes, *FvMYB10 *and *FaMYB10*, was examined by qPCR analysis during a fruit development series of wild diploid strawberry (*Fragaria vesca*) and cultivated octaploid strawberry (*Fragaria *× *ananassa*; Figure 7). Expression of an R2R3 MYB repressor of anthocyanin biosynthesis, *FaMYB1 *[19] was also examined in the same fruit series. There was a large increase in the relative transcript levels of the *MYB10 *transcription factor in the fruit tissues (Figure 7A). In *F. ananassa*, transcript levels of *FaMYB10 *were detectable but low until fruit were full size (Figure 7B). Upon ripening and colour change, there was an almost 40,000-fold increase in relative transcript level. *FaMYB1 *showed an expression pattern similar to that published, with the highest transcription level at the ripe fruit stage [19] while *FvMYB1 *expression showed little change. Expression levels of *FvMYB10 *in *F. vesca *also correlate with colour change. *F. vesca *has an earlier colour change, which occurs only in the skin (Figure 7C). For the mature fruit, the increase of *FaMYB10 *is almost 10 times more than that of *FvMYB10*. This may be due to cultivated strawberry fruit having anthocyanin throughout fruit flesh and skin while the wild strawberry accumulates anthocyanin only in the outer cell layers of the mature fruit.

Under stressful conditions (high light), the petals of *F. vesca *flowers became pigmented (Figure 7D). While *FvMYB1 *showed little change in these petals, the transcript of *FvMYB10 *from this tissue showed a large increase in accumulation compared with the petals that were not exposed to high light and were unpigmented. This is further evidence that MYB10 in strawberry is involved in regulating anthocyanin accumulation.

### Transformation of MYB10 into the crop of origin results in elevation of anthocyanin biosynthesis

**Figure 8:**
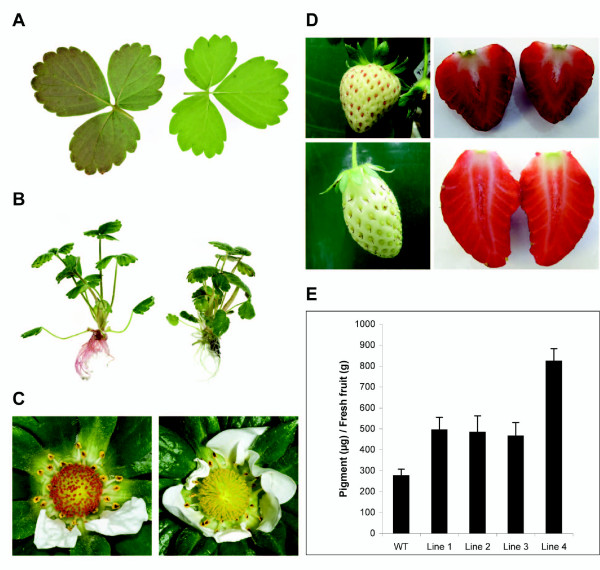
**Transformation of strawberry with 35S:*FaMYB10 *elevates anthocyanin synthesis**. Cultivated strawberry was transformed with 35S:*FaMYB10*. Visible reddening was seen in leaves (A; WT on right) and roots (B; WT on right), and in flowers (C; WT on right). Fruits showed red seeds and elevated anthocyanin (D; transgenic top photos, WT below). Extracted pigment was anthocyanin and increased in all lines (E). Error bars are the SE for four replicate extracts per line.

It has been recently reported that transformation of 'Royal Gala' apple with 35S:*MdMYB10 *results in plants ectopically accumulating anthocyanins [24,42]. In contrast, when 35S:*FaMYB10 *was transformed into *F. ananassa*, (using an adapted protocol [47]), callus and plantlets were not highly pigmented. When these plants were grown under short day conditions (8 h day, 16 h night) to encourage flowering and then transferred to long days, 35S:*FaMYB10 *plants had elevated foliar anthocyanins (Figure 8A), and red roots (Figure 8B). All of the 35S:*FaMYB10 *transgenic lines had flowers which showed distinctive red stigmas (Figure 8C). Transgenic fruit from these lines had immature fruits with red seeds, and mature fruits with approximately 50% more anthocyanin. These fruit had the same compound profile as wild-type fruit (cyanidin-glucoside: pelargonidin-glucoside: pelargonidin rutinoside at approximately 1:50:5 as measured with HPLC; Figure 8E, Additional File 3). Transcript analysis of 35S:*FaMYB10 *lines confirmed an elevation of *FaMYB10 *transcript level in both the fruit and leaf tissue (Additional File 3). No elevation in *FaMYB1 *transcript level was observed in transgenic tissue versus wild-type.

## Discussion

### The plant MYB family

The MYB TF superfamily illustrates how a relatively small family in animal genomes (3 members of this TF type in the human genome by BLAST match) controlling cell division and differentiation has become the most abundant TF group in plants [48] with diverse functions in hormone response [49], growth [50], epidermal cell fate and formation of trichomes [51], stomatal movements and development [52]; [53], seed development [54], response to drought [55] and cold [56,57], pathogen-response [58,59], light-sensing responses [60,61], sugar-related responses [62], modulation of secondary metabolites such as glucosinolates [63,64] and phenylpropanoids [65]. MYB proteins have a conserved N-terminal DNA binding domain of 100-160 residues, depending on the number of R repeats, with each repeat containing a helix-helix-turn-helix structure. Within this N-terminal region are key residues important for trans-activation efficiency [66], residues that regulate and specify DNA binding [14], and interactions with bHLHs [67]. We have identified in this study several residues shared by anthocyanin-promoting MYBs, from diverse species, that may be important in their function (Figure 4).

Consensus motifs in the C-terminus of MYBs, important for function are just beginning to be elucidated. One such example is the case of the C2 EAR motif repressor clade. AtMYB4 has the motif NLELRISLPDDV, which is essential for its repressive activity against the CH4 promoter [20]. This motif (pdLNLD/ELxiG/S) is also conserved in a number of R2R3 MYB proteins belonging to subgroup 4 which includes AtMYB4, AtMYB6, AtMYB7 and AtMYB32, and *Antirrhinum *AmMYB308 and AmMYB330, which have very similar effects to AtMYB4 when over-expressed in tobacco [21]. FaMYB1 also has such a motif [19]. In anthocyanin-promoting MYBs, the motif KPRPR[S/T]F was identified [65]. By analysing more MYBs of this clade we found variation in this C-terminal motif (Figure 2), but enough conservation to suggest it could be used as an identifier.

### MYBs involved in regulation of phenylpropanoid levels

The phenylpropanoids include flavonoids, anthocyanins, and proanthocyanidins. The accumulation of these compounds in plants and plant organs is central to such quality parameters as colour, human health, bitterness and astringency, as well as plant response to biotic and abiotic stress. R2R3 MYBs are responsible for controlling different aspects of the phenylpropanoid pathway in a wide range of different plant species. These include flavonol-specific MYBs [65], proanthocyanidin-specific MYBs [68], inhibitors of branch points [69] and R2R3 MYBs specifically controlling the anthocyanin biosynthetic pathway genes as well as anthocyanin conjugation, transport into the vacuole [70], and acidification of this compartment to affect fruit/flower/foliage colour [71].

In *Arabidopsis*, one of the R2R3 anthocyanin-related clades is made up of AtMYB75 (PAP1, At1g56650; [18], AtMYB90 (AtPAP2, At1g66390), AtMYB113 (At1g66370), and AtMYB114 (At1g66380). As three of these MYBs occur in order on chromosome 1, they may have arisen by tandem duplications of AtMYB75. Over-expression of PAP1 [70], AtMYB113 and AtMYB114 [26] all result in elevated anthocyanin levels. By examining homologues of PAP1 in other species, we have identified residues that predict MYBs involved in anthocyanin regulation. This anthocyanin-promoting clade is apparently absent in the rice genome and other monocots and gymnosperms, suggesting recent divergence of these MYBs.

In apple, three MYB genes have been independently isolated, all of which control anthocyanin levels and show a very high degree of sequence similarity; *MYB10*, *MYB1 *and *MYBA*. It has been suggested that *MYB1 *and *MYBA *are alleles arising from the different varieties from which they were cloned [38]. The *MYB10 *sequence is more diverse. It is difficult, from sequence analysis alone, to distinguish between recently duplicated gene paralogues and allelic variation between different varieties. By designing PCR primers to a region of sequence common to both *MYB10 *and *MYB1*, we were able to distinguish between the *MYB10 *allele from 'Red Field', which produces a 1000-bp amplification product and other *MYB10 *alleles (from 'Royal Gala' amongst others) and the *MYB1 *alleles from Pacific Rose™ and 'Royal Gala' that produce a 900-bp fragment. If *MYB1 *and *MYB10 *were paralogues then, in the varieties that only amplified a 900-bp fragment, this fragment would be the product of four alleles (i.e. two from each of the parologous genes). However, if *MYB10 *and *MYB1 *are allelic, the 900-bp fragment would only be produced by two alleles, one from each of the *MYB10/MYB1 *alleles of the parents. Accordingly, in red-fleshed varieties which are heterozygous for the promoter polymorphism, such as 'Robert's Crab', one of the *MYB10 *alleles produces a 1000-bp fragment, and the other allele a 900-bp fragment. In homozygous *Malus sieversii *01P22 there is only the 1000-bp fragment. In addition to this, if *MYB1 *was a paralogue, a further two 900-bp products would be contributed from the *MYB1 *alleles. As we do not see DNA fluorescence consistent with a 1:3 amplification of the 1000- and 900-bp fragments, we propose that *MYB10 *and *MYB1/MYBA *are alleles. The future availability of whole genome sequence for apple will aid a conclusion on the allelic structure of this gene.

### Identification of anthocyanin-promoting MYB10 genes in rosaceous crops

Using degenerate PCR based on the *MYB10 *sequence, we have been able to isolate 20 MYB10-like genes from a range of rosaceous species. Analysis of the genomic DNA of these species predicts that all the genes contain two introns in positions consistent with the intron location of other *MYB *genes [72]. Almost all of the variation in gene size is due to alterations in the predicted length of intron 2 (Table 1). Aligning intron 2 of *Malus MYB10 *(crab and domesticated apple; ~3000 bp) and *Pyrus MYB10 *(European and Asian pears; ~500 bp) revealed a high degree of similarity except within a region of intron 2 where there appears to have been a 2500-bp insertion. This does not contain inverted repeats or sequence signatures that are indicative of mobile genetic elements such as transposons or helitrons [73]. This insertion could be the result of a local genome rearrangement that took place after the speciation of apple and pear, but before the divergence of apple and crab apples.

Botanical classification of the Rosaceae has recently been undertaken using 88 species analysed for a combination of phenotypic and molecular marker [74]. Using the nuclear encoded genes, polygalacturonase inhibitor protein (PGIP) and polyphenol oxidase (PPO), a weak or conflicting phylogenic resolution was produced. We have complemented this analysis, on a smaller dataset, by adding additional information relating to a single copy nuclear gene, where orthology has been inferred by both sequence and functional characterisation. The phylogenic placements are in broad agreement with the *Pyrinae *sub-tribe and *Amygdaloideae *tribe described [74].

### Transient activation of the anthocyanin pathway by rosaceous MYB10s requires a bHLH

Within the R2R3 domain of all 20 MYB10s there were several key motifs suggesting an association with a bHLH partner. Several anthocyanin-promoting MYBs have been assayed in heterologous systems; for example, tobacco [24,39], *Arabidopsis *[39] or *Antirrhinum *petal cells [34] and this trans-activation is often enhanced by the co-infiltration of an appropriate *bHLH *gene [24,37,42]. We used transient assay of rosaceous MYB genes, with the DFR promoter from *Arabidopsis *and bHLH genes from either apple or *Arabidopsis*. All of the isolated MYB TFs were able to trans-activate the *DFR *promoter in the presence of at least one of the four bHLHs tested. Only the apple MYB10 gene responded equally to either apple or *Arabidopsis *bHLH genes. This may indicate a degree of specificity that exists for the apple bHLH gene and its association with apple MYB gene and target promoter. However, the degree of trans-activation, and interaction with bHLH partners, varies greatly amongst the rosaceous MYBs genes tested (Figure 5). In particular, plum and apricot showed trans-activation values in excess of five times that of the 35S-promoter. Such high trans-activation potential may be due to more effective interaction of plum and cherry MYBs with tobacco transcription factors endogenous within the transient assay, or could point to an enhanced ability of these MYBs to promote high levels of anthocyanin. Further analysis of these MYB TFs in homologous systems is required, and techniques such as yeast-2-hybrid used to probe which protein residues are responsible for strong or weak interactions.

### MYB10 expression is strongly associated with anthocyanin production in fruits

During fruit development, in both strawberry and cherry, the transcript level of *MYB10 *was up-regulated. A correlation between transcript and anthocyanin production has already been reported in apple [24,38,39]. In a cherry cultivar which has lower anthocyanin levels at maturity the expression of *MYB10 *transcript was lower than in a dark-fruited cultivar. It remains for these genes to be mapped in crops segregating for different pigmentation levels. However, for apple, *MYB10/MYB1/MYBA *is the major gene in a crossed population segregating for red flesh [75] and red skin [38].

Transformation of strawberry with *FaMYB10 *resulted in plants with elevated root, foliar and fruit anthocyanin levels (Figure 8). These levels were not as high as previously reported in 35S:*MdMYB10 *apple transformants [24], due perhaps to other partners in the MYB/bHLH/WD40 complex. It has been shown recently that tomato fruits, with elevated anthocyanins due to over-expression of MYB and bHLH members of the MBW complex, are responsible for promoting human health attributes [6,76].

## Conclusions

The Rosaceae family-wide characterisation of MYBs provides insight into the evolution of this TF and has implications for the understanding of temporal-spatial colour change. Our identification of this set of MYBs will aid development of new rosaceous fruit and flowers by allowing the testing of co-segregation of MYB alleles with pigment phenotypes in Rosaceae, which are both common and highly sought after (e.g., rose, plum, cherry, peach). If these candidate genes do segregate for anthocyanin levels, gene-based marker-assisted selection or even cisgenics could be used in breeding programmes. This approach has worked for apple [75] and there is preliminary evidence that PavMYB10 co-locates with a QTL for fruit and flesh colour in cherry (A. Iezzoni and J. Bushakra, pers. comm.) and PprMYB10 co-locates with anther colour segregating in the peach reference map (J. Bushakra and P. Arus, pers. comm.). This group of transcription factors therefore becomes useful as a breeding and biotechnological tool.

## Methods

### Isolation of rosaceous transcription factors

Fruit and leaf samples of 20 rosaceous species were collected as follows: crab apple (*Malus sylvestris*), sweet cherry (*Prunus avium*), sour cherry (*Prunus cerasus*), almond (*Prunus dulcis*), peach (*Prunus persica*), Japanese plum (*Prunus salicina*) and rose (*Rosa hybrida*) from Auckland Botanic Gardens (Auckland, New Zealand); quince (*Cydonia oblonga*), loquat (*Eriobotrya japonica*), medlar (*Mespilus germanica*), pear (*Pyrus communis*), apricot (*Prunus armeniaca*), Damson (*Prunus insititia*), European plum (*Prunus domestics*), cherry-plum (*Prunus cerasifera*) from local gardens (Auckland, New Zealand); strawberry (*Fragaria *× *ananassa *and *Fragaria vesca*) from Plant & Food Research greenhouse (Auckland, New Zealand); red raspberry 'Latham' (*Rubus idaeus*), pear 'Nashi' (*Pyrus pyrifolia*), and Chinese pear 'Yali' (*Pyrus *× *bretschneideri*) from various Plant & Food Research orchards.

Messenger RNA (mRNA) was isolated using an adapted method [77] from pigmented fruit or flower tissue, and genomic DNA (gDNA) was isolated (DNeasy Plant Mini Kit, Qiagen) from young leaves or flower buds. *MYB10*s of pear (*Pyrus communis*) and cherry-plum (*Prunus cerasifera*) were successfully obtained by applying various primers based on *MdMYB10 *in cDNA or gDNA PCR amplification. With more primers based on *PcMYB10 *and *PcfMYB10*, *MYB10*s from two subfamilies, *Maloideae *and *Amygdaloideae*, were completed by overlapping PCR fragments. For the subfamily *Rosoideae*, which includes *Fragaria, Rubus *and *Rosa*, degenerate primers, designed to the consensus DNA sequence of R2R3 binding domain were used in 5' and 3' GeneRace (GeneRacer Kit, Invitrogen). The complete sequence for *MYB10 *was compiled from overlapping fragments and full length clones were isolated using gene-specific primers designed to the 5' and 3' UTR regions. Phylogenic trees were generated using MEGA 3.1, a minimum evolution phylogeny test and 1000 bootstrap replicates.

### Dual luciferase assay of transiently transformed Nicotiana benthamiana leaves

The promoter of *Arabidopsis *DFR (*TT3*, AT5g42800) was isolated from genomic *Arabidopsis *DNA and cloned into pGreenII 0800-LUC vector [46]. *MYB10 *cDNA or gDNA full length sequence from 10 selected rosaceous species was cloned into pGreen II 62-SK 0029 binary vectors [46].

*Nicotiana benthamiana *plants were grown under glasshouse conditions until about 5 cm in height. Approximately 150 μl of *Agrobacterium *culture was infiltrated at four points into a young leaf. Three days after inoculation, 3-mm leaf discs (4 technical replicates from each plant) were cut with a hole-puncher, placed into wells of a 96-well-plate containing 50 μl of PBS (phosphate buffered saline) in each well, and gently crushed with the hole-puncher. The measurement and analysis was carried out using an Orion Microplate Luminometer (Berthold Detection System), using the manufacturer's recommended conditions.

### PCR expression analysis

Strawberry fruits from *Fragaria *× *ananassa *and *Fragaria vesca *were collected at six time points during fruit development: stage 1, pre-opened bud; stage 2, fully open flower; stage 3, petal drop; stage 4, expanding fruitlet; stage 5, expanded fruit; stage 6, red-ripe fruit, from plants grown under glasshouse conditions, using natural light with daylight extension to 16 h. In one instance, *Fragaria vesca *was grown under constant lighting, inducing red pigmented petals. RNA was isolated [77] from fruit (six samples from the same plant, skin and cortex combined), and red and white petals. First strand cDNA synthesis was carried out by using oligo dT according to the manufacturer's instructions (SuperScript III, Invitrogen). As the identity between cDNA sequences of *Fragaria *× *ananassa *and *Fragaria vesca *is as high as 95%, a set of qPCR primers was designed for both species using Vector NTI to a stringent set of criteria, enabling application under universal reaction conditions.

To eliminate gDNA contamination, both forward and reverse primers were designed to span an intron/exon boundary. The strawberry actin primers were based on the actin sequence of *Fragaria *× *ananassa *(Genebank number AB116565). The reverse primer of actin was also designed to span an intron.

Sweet cherry (*Prunus avium*) 'Stella' and 'Rainier' were collected at three time points during fruit development: stage 1, green fruitlet; stage 2, expanding fruit; stage 3, mature fruit, from a Plant & Food Research orchard (Clyde, New Zealand). Actin primers were based on the actin sequence of closely related sour cherry *Prunus cerasus *(Genebank number EE488162). The method of RNA extraction and the principles of primer design were the same as strawberry.

Quantitative real time PCR (qPCR) DNA amplification and analysis was carried out using the LightCycler System (Roche LightCycler 1.5, Roche), with LightCycler software version 4. The LightCycler FastStart SYBR Green Master Mix (Roche) was used, and the 10 μl of total reaction volume applied in all the reactions following the manufacturer's method. qPCR conditions were 5 min at 95°C, followed by 40 cycles of 5 s at 95°C, 5 s at 60°C, and 10 s at 72°C, followed by 65°C to 95°C melting curve detection. The qPCR efficiency of each gene was obtained by analysing the standard curve of a cDNA serial dilution of that gene. The expression was normalized to *Fragaria *× *ananassa *actin and *Prunus cerasus *actin with *Fragaria vesca *stage 1 flower bud and *Prunus avium *'Rainier' stage 1 fruitlet acting as calibrator with a nominal value of 1. Actin was selected as a reference gene because of its consistent transcript level throughout fruits and leaves. To confirm the amplification of the expected DNA sequence, qPCR amplicons were sequenced.

Endpoint PCR analysis used in the apple MYB allele study was carried out using Platinum Taq (Invitrogen). Reaction conditions were 95°C, 5 min followed by 35 cycles of 30 s at 95°C, 30 s at 55°C, and 60 s at 72°C. PCR products were separated on 1% agarose gels and stained with ethidium bromide. Primer sequences are listed in Additional File 4.

### Growth of Strawberry plants and Generation of 35S:*FaMYB10 Fragaria *× *ananassa *plants

Strawberry plants of *Fragaria *× *ananassa *and *Fragaria vesca *were grown under controlled conditions (23°C day, 15°C night) in a short day room (8 h day, 16 h night) for 3 months, then plants were moved to long day conditions (16 h day, 8 h night, 25°C day, 15°C night) to encourage flowering.

Surface sterilized seeds were germinated on 1/2 MS basal salt and vitamins (Duchefa) + 3% sucrose + 0.7% agar (Germantown) (pH 5.7) medium. Seedlings were sub-cultured onto fresh medium every four weeks. Young leaves excised from *in vitro *grown shoots were cut into ~1 × 2 mm leaf strips. Transformation was via an adapted protocol (from [47] with *Agrobacterium tumefaciens *strain EHA105 [78], harbouring the binary plasmid pGreen II 0029 62-sk [79] containing the NOS/NPT II for kanamycin resistance, and a CaMV 35s promoter-driven full length *FaMYB10 *cDNA.

### HPLC measurement of strawberry fruits

Two mature strawberry fruits were taken from each of three plants representing two transgenic lines and a wild-type control. The fruits were freeze-dried for at least 24 h. The dried tissue was then pulverized, resuspended in ethanol: distilled water: formic acid (80:20:1) with the ratio of 5 mL solvent to 1 g of original fresh fruit weight, extracted at room temperature for 3 h in the dark, centrifuged at 3500 rpm for 10 min. A 1-ml aliquot of the supernatant was analyzed for anthocyanin components by HPLC. The HPLC system consisted of a Waters Alliance Separation Module (model 2690) and a photodiode array detector (model 996) under the control of Chromeolen^® ^(Dionex, USA) software. The separation column used was a Zorbax Rapid Resolution SB-C18 4.6 × 150 mm (Agilent, USA) with a binary solvent program (A = formic acid/MQ water (5:95); B = acetonitrile) that started at 95% A 5% B at injection, changed to 80%A 20%B at 9 minutes; 20%A 80%B at 18 minutes and held for 2 minutes before returning to 95%A 5%B ready for the next sample injection. Total flow rate was 0.8 mL/min and sample injection volumes were 5 μL. Anthocyanin components were detected at 530 nm, and peaks indentified by retention time with authentic standards, and previous reports of strawberry anthocyanins.

## Authors' contributions

KLW, KB, KG and AK isolated and cloned the rosaceous MYBs. AK and RVE designed and performed allele specific apple gene amplification. SK cloned bHLH transcription factors. KLW transformed strawberry, and TKM analyzed the resulting plants. KLW, RPH, and ACA conceived the study, participated in the design, and drafted and edited the manuscript. All authors read and approved the final manuscript.

## Supplementary Material

Additional file 1 **Schematic of the *MYB10 *gene from all the major rosaceous species**. MYB10 exon and intron composition, with the size of intron 2 variation as a correlation with estimated genome size. Click here for file

Additional file 2 **Table of key amino-acid residues in R2R3 MYBs**. Key amino-acid motif at position 90 to 93 in R2R3 domain of 173 MYB transcription factors of *Arabidopsis*, Rosaceae, and other species. Click here for file

Additional file 3 **Analysis of transgenic strawberry**. qPCR of *MYB10 *and *MYB1 *and extracted anthocyanins of wild type ripe fruit and 35S-*MYB10 *ripe fruit. Click here for file

Additional file 4 **Primers used in this study**. Table of oligonucleotide primers used in this study. Click here for file
