# Supplementary material for: An R2R3 MYB transcription factor associated with regulation of the anthocyanin biosynthetic pathway in Rosaceae
Source: BMC Plant Biol. 2010 Mar 21;10:50. doi: 10.1186/1471-2229-10-50 (PMC2923524; doi:10.1186/1471-2229-10-50)
Supplement: Additional file 1 — Schematic of the MYB10 gene from all the major rosaceous species. MYB10 exon and intron composition, with the size of intron 2 variation as a correlation with estimated genome size. [file 1471-2229-10-50-S1.PPT]

## Slide 1
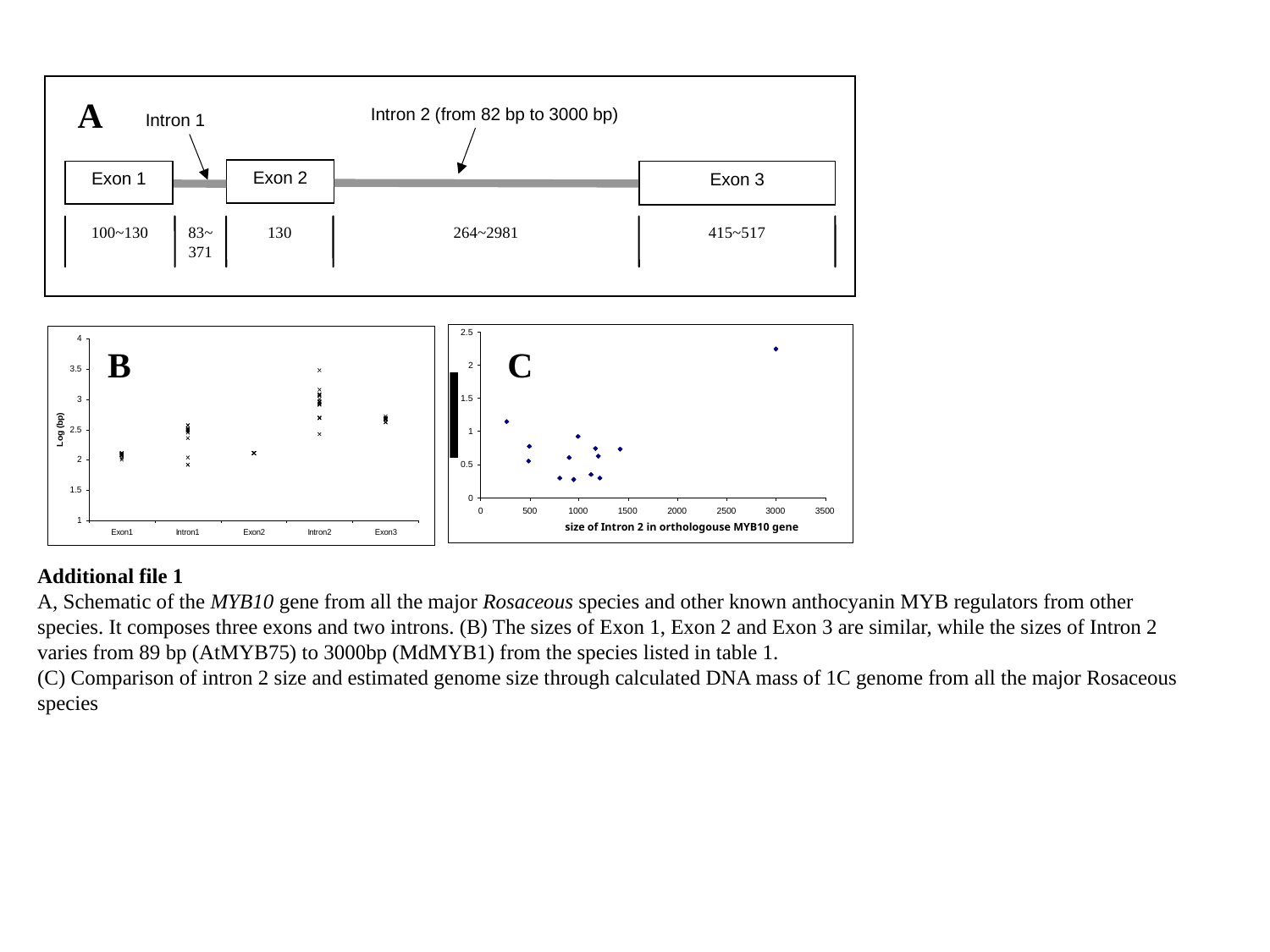

A
Intron 2 (from 82 bp to 3000 bp)
Intron 1
Exon 2
Exon 1
Exon 3
100~130
83~
371
130
264~2981
415~517
2.5
2
1.5
1
0.5
0
0
500
1000
1500
2000
2500
3000
3500
size of Intron 2 in orthologouse MYB10 gene
B
C
Additional file 1
A, Schematic of the MYB10 gene from all the major Rosaceous species and other known anthocyanin MYB regulators from other species. It composes three exons and two introns. (B) The sizes of Exon 1, Exon 2 and Exon 3 are similar, while the sizes of Intron 2 varies from 89 bp (AtMYB75) to 3000bp (MdMYB1) from the species listed in table 1.
(C) Comparison of intron 2 size and estimated genome size through calculated DNA mass of 1C genome from all the major Rosaceous species
