# Supplementary material for: An R2R3 MYB transcription factor associated with regulation of the anthocyanin biosynthetic pathway in Rosaceae
Source: BMC Plant Biol. 2010 Mar 21;10:50. doi: 10.1186/1471-2229-10-50 (PMC2923524; doi:10.1186/1471-2229-10-50)
Supplement: Additional file 2 — Table of key amino-acid residues in R2R3 MYBs. Key amino-acid motif at position 90 to 93 in R2R3 domain of 173 MYB transcription factors of Arabidopsis, Rosaceae, and other species. [file 1471-2229-10-50-S2.PPT]

## Slide 1
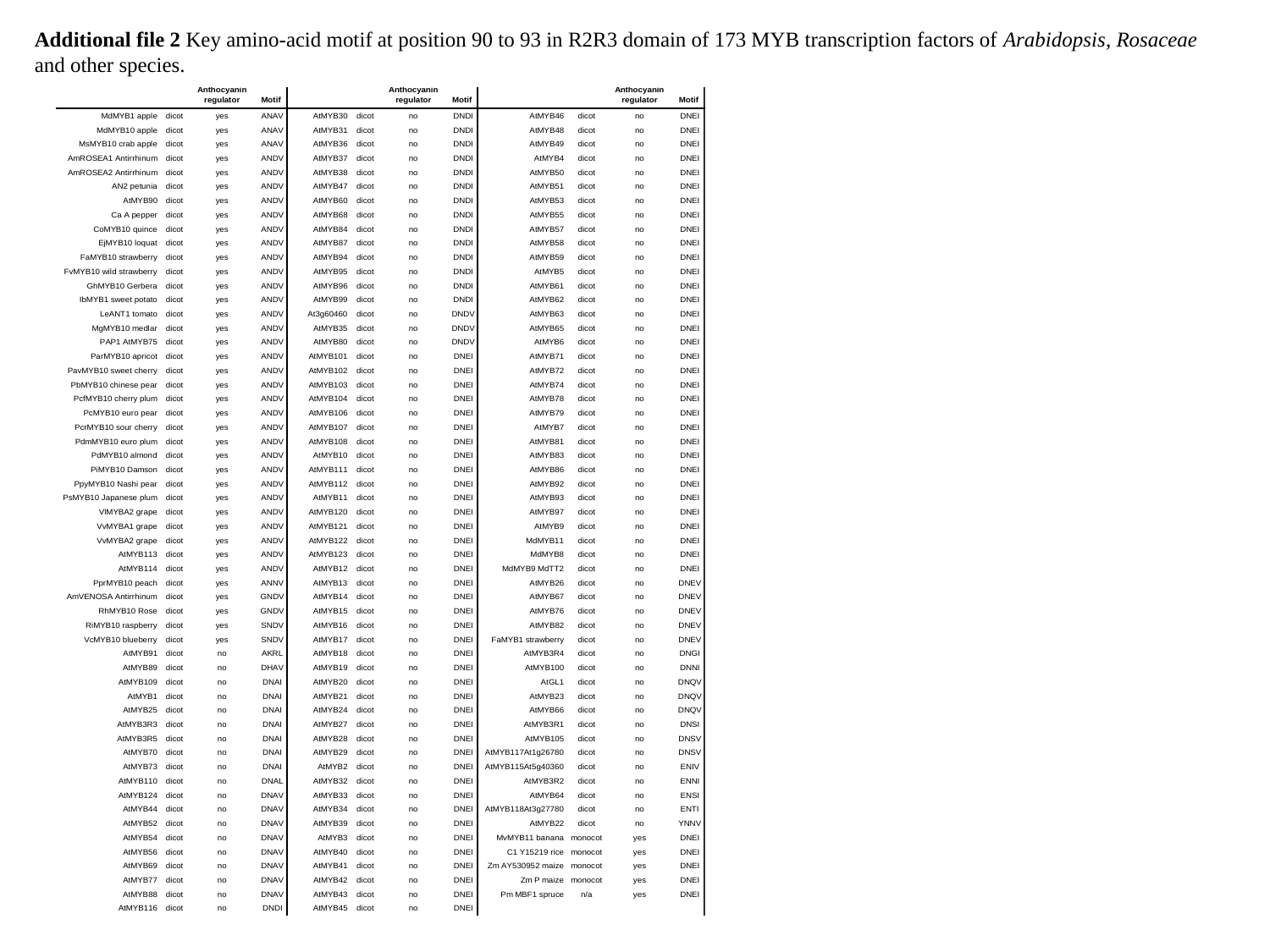

Additional file 2 Key amino-acid motif at position 90 to 93 in R2R3 domain of 173 MYB transcription factors of Arabidopsis, Rosaceae and other species.
