# Supplementary material for: An R2R3 MYB transcription factor associated with regulation of the anthocyanin biosynthetic pathway in Rosaceae
Source: BMC Plant Biol. 2010 Mar 21;10:50. doi: 10.1186/1471-2229-10-50 (PMC2923524; doi:10.1186/1471-2229-10-50)
Supplement: Additional file 3 — Analysis of transgenic strawberry. qPCR of MYB10 and MYB1 and extracted anthocyanins of wild type ripe fruit and 35S-MYB10 ripe fruit. [file 1471-2229-10-50-S3.PPT]

## Slide 1
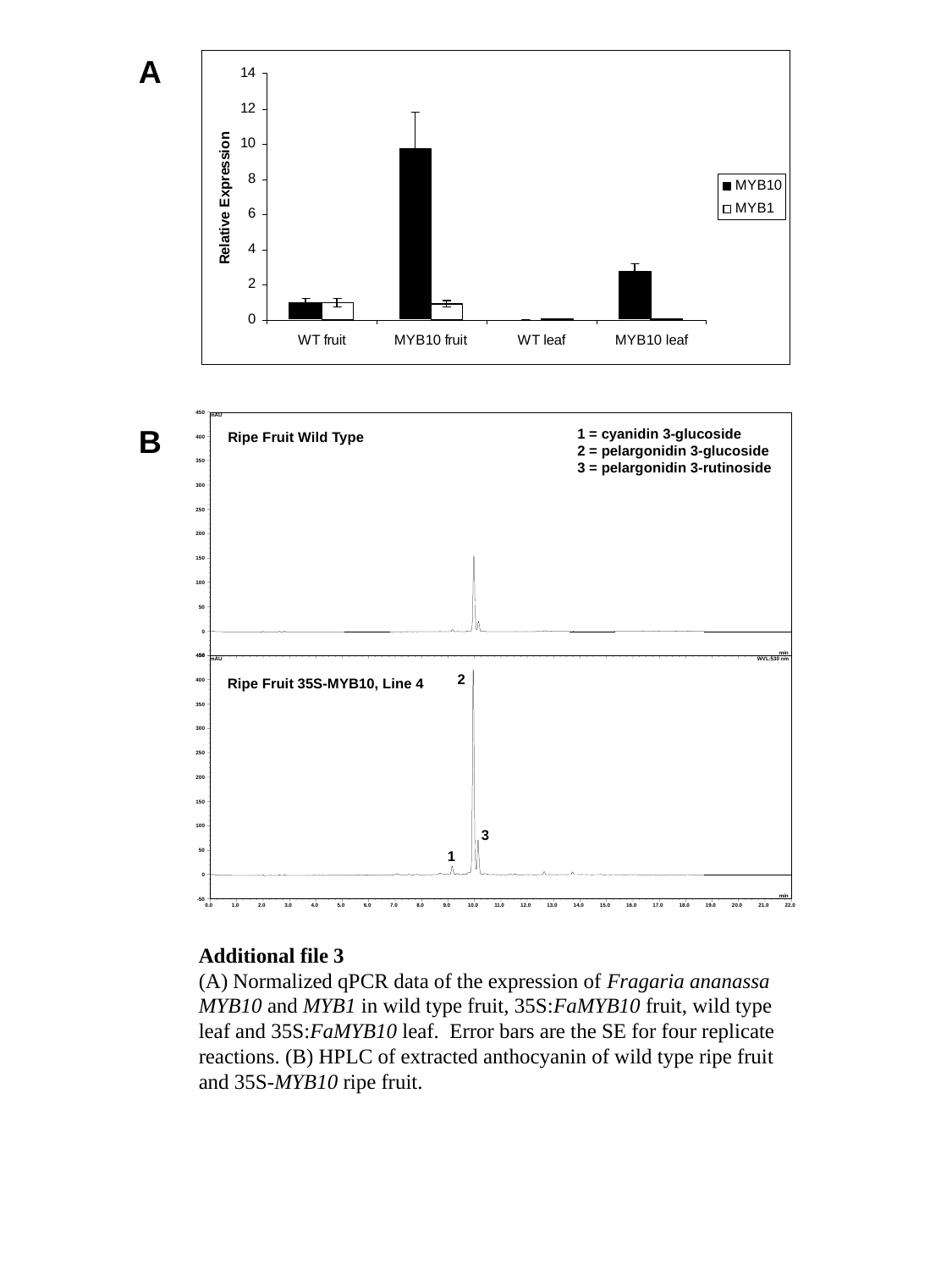

A
450
mAU
1 = cyanidin 3-glucoside
2 = pelargonidin 3-glucoside
3 = pelargonidin 3-rutinoside
Ripe Fruit Wild Type
400
350
300
250
200
150
100
50
0
450
mAU
WVL:530 nm
400
350
300
250
200
150
100
50
0
min
-50
0.0
1.0
2.0
3.0
4.0
5.0
6.0
7.0
8.0
9.0
10.0
11.0
12.0
13.0
14.0
15.0
16.0
17.0
18.0
19.0
20.0
21.0
22.0
min
-50
2
Ripe Fruit 35S-MYB10, Line 4
3
1
B
Additional file 3
(A) Normalized qPCR data of the expression of Fragaria ananassa MYB10 and MYB1 in wild type fruit, 35S:FaMYB10 fruit, wild type leaf and 35S:FaMYB10 leaf. Error bars are the SE for four replicate reactions. (B) HPLC of extracted anthocyanin of wild type ripe fruit and 35S-MYB10 ripe fruit.
