# Supplementary material for: An R2R3 MYB transcription factor associated with regulation of the anthocyanin biosynthetic pathway in Rosaceae
Source: BMC Plant Biol. 2010 Mar 21;10:50. doi: 10.1186/1471-2229-10-50 (PMC2923524; doi:10.1186/1471-2229-10-50)
Supplement: Additional file 4 — Primers used in this study. Table of oligonucleotide primers used in this study. [file 1471-2229-10-50-S4.PPT]

## Slide 1
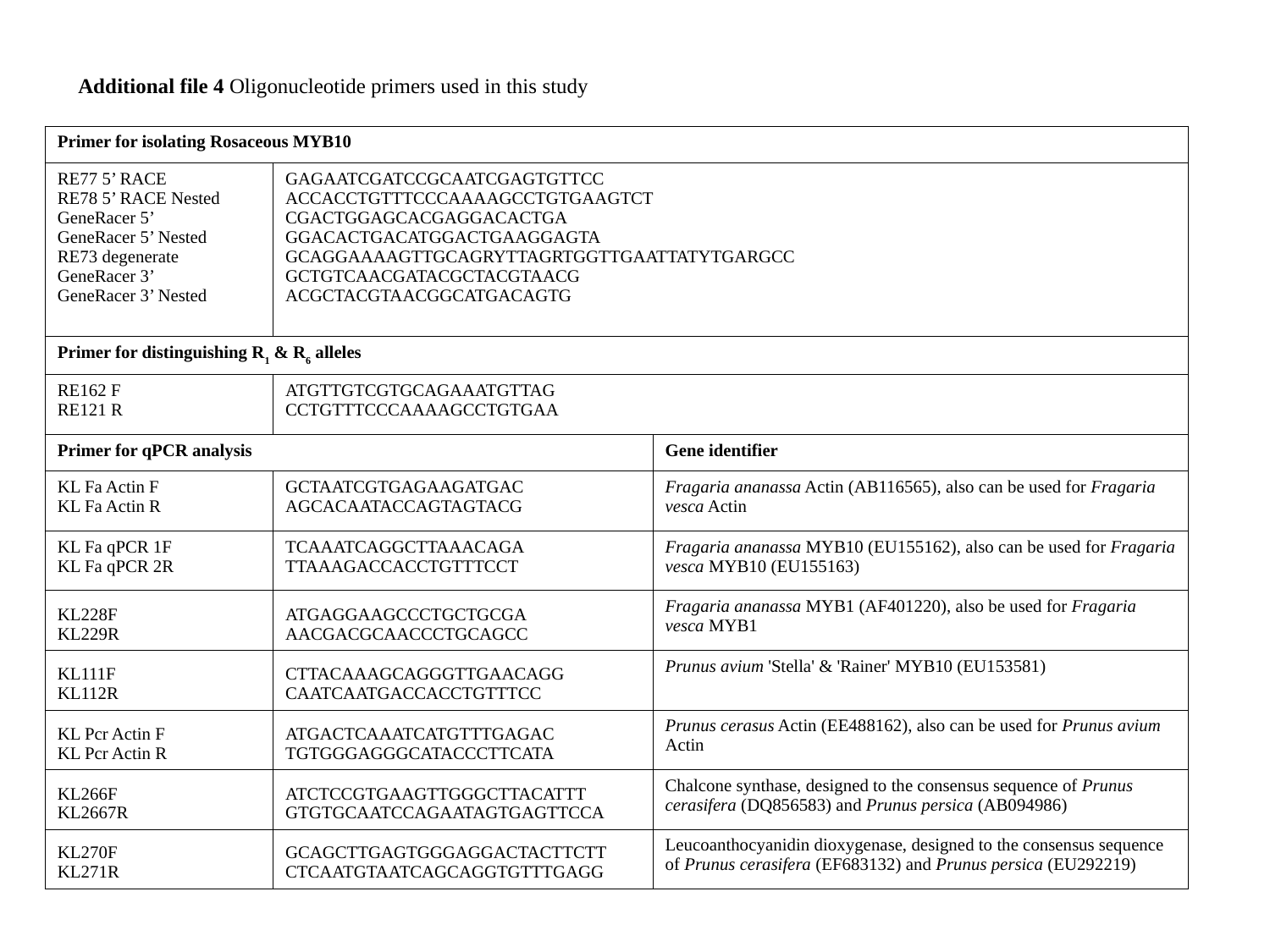

Additional file 4 Oligonucleotide primers used in this study
| Primer for isolating Rosaceous MYB10 | | |
| --- | --- | --- |
| RE77 5’ RACE RE78 5’ RACE Nested GeneRacer 5’ GeneRacer 5’ Nested RE73 degenerate GeneRacer 3’ GeneRacer 3’ Nested | GAGAATCGATCCGCAATCGAGTGTTCC ACCACCTGTTTCCCAAAAGCCTGTGAAGTCT CGACTGGAGCACGAGGACACTGA GGACACTGACATGGACTGAAGGAGTA GCAGGAAAAGTTGCAGRYTTAGRTGGTTGAATTATYTGARGCC GCTGTCAACGATACGCTACGTAACG ACGCTACGTAACGGCATGACAGTG | |
| Primer for distinguishing R1 & R6 alleles | | |
| RE162 F RE121 R | ATGTTGTCGTGCAGAAATGTTAG CCTGTTTCCCAAAAGCCTGTGAA | |
| Primer for qPCR analysis | | Gene identifier |
| KL Fa Actin F KL Fa Actin R | GCTAATCGTGAGAAGATGAC AGCACAATACCAGTAGTACG | Fragaria ananassa Actin (AB116565), also can be used for Fragaria vesca Actin |
| KL Fa qPCR 1F KL Fa qPCR 2R | TCAAATCAGGCTTAAACAGA TTAAAGACCACCTGTTTCCT | Fragaria ananassa MYB10 (EU155162), also can be used for Fragaria vesca MYB10 (EU155163) |
| KL228F KL229R | ATGAGGAAGCCCTGCTGCGA AACGACGCAACCCTGCAGCC | Fragaria ananassa MYB1 (AF401220), also be used for Fragaria vesca MYB1 |
| KL111F KL112R | CTTACAAAGCAGGGTTGAACAGG CAATCAATGACCACCTGTTTCC | Prunus avium 'Stella' & 'Rainer' MYB10 (EU153581) |
| KL Pcr Actin F KL Pcr Actin R | ATGACTCAAATCATGTTTGAGAC TGTGGGAGGGCATACCCTTCATA | Prunus cerasus Actin (EE488162), also can be used for Prunus avium Actin |
| KL266F KL2667R | ATCTCCGTGAAGTTGGGCTTACATTT GTGTGCAATCCAGAATAGTGAGTTCCA | Chalcone synthase, designed to the consensus sequence of Prunus cerasifera (DQ856583) and Prunus persica (AB094986) |
| KL270F KL271R | GCAGCTTGAGTGGGAGGACTACTTCTT CTCAATGTAATCAGCAGGTGTTTGAGG | Leucoanthocyanidin dioxygenase, designed to the consensus sequence of Prunus cerasifera (EF683132) and Prunus persica (EU292219) |
